# Ears Wide Shut: A Complete Failure to Recognize Relevant Acoustic Stimuli

**DOI:** 10.21203/rs.3.rs-3131628/v1

**Published:** 2023-07-24

**Authors:** Robert Stickgold, Cynthia Okamoto, Katrina Hon, Dan Denis

**Affiliations:** Harvard Medical School; Beth Israel Deaconess Medical Center; Beth Israel Deaconess Medical Center; University of York

## Abstract

The filtering out of apparently extraneous and redundant stimuli is critical for the effective processing of novel and relevant sensory information. But brain mechanisms that evolved to perform this function are necessarily less than perfect, in some cases failing to filter out irrelevant stimuli and in others filtering out important information. We report here on a stimulus from everyday life—the sound made by an arriving elevator, which contains information indicating the car’s direction of movement—that not one of over 1,100 study participants was aware of, despite encountering this information repeatedly throughout their lives. Evidence of implicit knowledge of this information was also absent, suggesting that this valuable information is filtered out at an early stage of sensory processing.

## Introduction

Consider the Elevator Dilemma: How do blind people, waiting for an elevator, determine, without help from others, which way it is going when it arrives? Most readers will struggle to answer this question, despite an answer that they should already be aware of: The Americans with Disabilities Act of 1990 (U.S.) requires all elevators to produce “audible signals [that] sound once for the up direction and twice for the down direction “ (1, *p 135*). Yet, in the study we report here, not one of the 1,173 participants knew the significance of the second ‘ding’ sounded by arriving elevators.

## Results

### Experiment One: Explicit knowledge of the elevator rule

In our first experiment, we used Amazon Mechanical Turk (2) to ask 1,039 participants the following question: “Imagine that you’re in a building, waiting by yourself for an empty elevator while blindfolded. Without removing the blindfold or getting on the elevator, would you be able to tell whether an arriving elevator was going up or down?” For those responding “yes”, we asked how they would do so, and then, after they responded, gave them a list of six possible answers and asked them to select the one closest to theirs. Those participants who indicated that they did not know how to solve this problem were given the same list of possibilities and asked to select which answer seemed most likely to work

**Figure 1:**
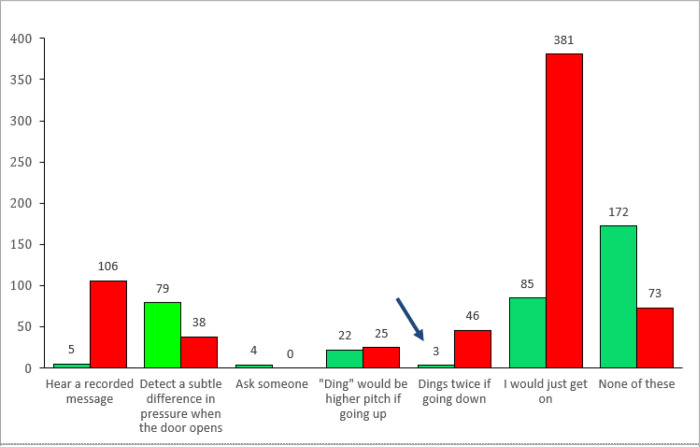
Distribution of selected answers to the Elevator Dilemma Question. Green: participants who indicated that they knew how to tell which direction an elevator was going; Red: participants indicating they did not know; Blue arrow: Only 3 participants indicated they knew how to tell and also selected the correct multiple-choice answer (dings twice if going down).

Overall, 370 participants (36%) responded that they knew how to determine the direction of the elevator, but only 3 of these (<1%) chose the correct answer from the list—the elevator “would ding twice if it were going down” (Fig. 1). Upon review of their written responses, it was clear that two of the three had not known the correct answer, while the third provided an ambiguous, but most likely incorrect, answer (“I recently learned that elevators make one tone for up and another for down”; Table 1). Among the 669 participants who reported that they did not have a solution for the dilemma, 46 (7%) selected the correct option from the list of six possibilities provided, less than half of what would have been expected by chance. Thus, none of the 1,039 subjects provided convincing evidence that they knew the solution to the Elevator Dilemma, a rate significantly less than one in 200 (binomial test, *p* = 0.04).

### Experiment 2: Implicit knowledge of the elevator rule

Despite their total lack of explicit awareness of the elevator rule, it remained possible that individuals had implicit knowledge of the meaning of the different sounds made by elevators. We investigated this in a second experiment involving 150 participants, of whom 114 completed the protocol. Participants were presented with recorded elevator sounds in two blocks of 16 trials each. For the first block, participants heard one ding on eight trials and two dings on the other eight. After each presentation, they were asked to indicate the elevator’s direction of travel and their confidence in their answer. In the second block, participants completed the same task but heard either a higher or lower pitch sound on each trial—a distinction that is not, in fact, ever used to indicate the direction of an elevators (1, *p 135*). At the end of the experiment, participants were asked to rate the accuracy of a series of statements about elevator sound-direction combinations (*e.g*., ‘one ding means up’ or ‘a higher pitch means down’) on a 1–8 scale ranging from completely false to completely true.

**Figure 2:**
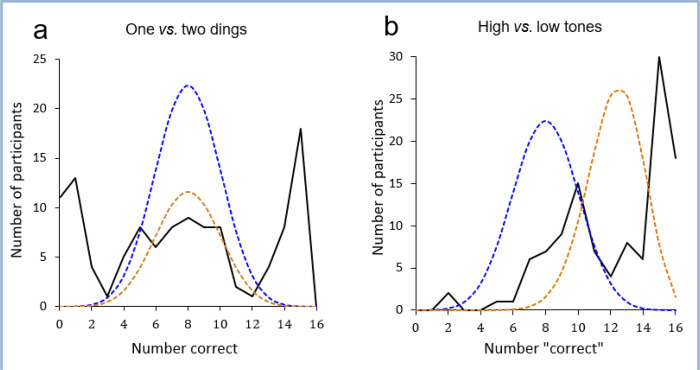
Distribution of participant scores. **A**:one *vs*. two dings. Solid line – distribution of participant scores; blue dashed line – random binomial distribution of all 114 scores; orange dashed line – random binomial distribution for *n* = *59* participants with scores of 4–13 correct. **B**: high *vs* low pitch tones. Solid line – distribution of participants based on number of trials scored as high = up or low = down; blue dashed line – random binomial distribution; orange dashed line – binomial distribution with 76.5% probability of high pitch = up or low pitch = down, matching actual percentage of trials similarly scored.

Overall, participants were at chance for correctly assigning direction in the 16 one- and two-ding trials (47.6% correct; *t* (114) = 0.80, *p* = 0.43). But a chi-square test comparing the symmetry of the distribution with the random binomial curve (Fig. 2A, **blue curve**) was significant (χ ² = 50.4, p < 0.0001). Indeed, despite this nearly 50–50 overall split on correct responses, 26 participants (23%) responded correctly on at least 14 (88%) of the 16 trials (binomial test, *p* ≤ 0. 002 for each participant) while an even larger number (*n* = 28, 25%) responded incorrectly on the same number of trials. Those who answered correctly on 3–13 trials (*n* = 60) appeared to have guessed randomly, approximating a binomial distribution (χ^2^ = 6.4, *p* = 0.78; Fig. 2A).

These differences were also reflected in participant’s judgements about sound-direction combinations at the end of the experiment. Participants who answered 14–16 trials correctly rated the statement “one ding means up” as significantly truer than those who answered approximately half (7–9 out of 16) correct (6.5 ± 0.3 vs. 4.0 ± 0.2, *df* = 50, *t* = 7.7, p < 0.0001). But the reverse was also true; participants who answered 14–16 trials incorrectly rated the statement “one ding means down” as significantly truer than participants who answered 7–9 incorrectly (5.5 ± 0.3 vs. 4.0 ± 0.2l, *df* = 52, *t* = 4.0, *p* = 0.0001).

In contrast, for the high vs. low pitch judgement, which, in fact, is not used to indicate elevator directions, 97 participants (85.1%) assigned an upward direction for the higher pitch sound on most (9–16) trials, while only 10 participants (8.8%) preferentially assigned an upward direction to the lower pitch tone (binomial test, *p* < 0.0001). In this case, only 2 participants (1.4%) scored 14–16 trials as “high = down” or “low = up”, whereas 54 participants (47%) scored a similar number of trials as “high = up” or “low = down” (Fig. 2B). Thus, the vast majority of participants (n = 97, 85%) drew the erroneous conclusion that higher pitched tones indicate an elevator going up and lower pitched tones indicate one going down. In subsequent evaluations, participants who scored at least 14 trials as high = up or low = down rated the truth of the statement “a high-pitched sound indicates up” higher than those who only scored about half (7–9) the trials in this manner (6.7 vs. 3.6, *df* = 75, *t* = 9.9, *p* < 0.0001).

**Figure 3:**
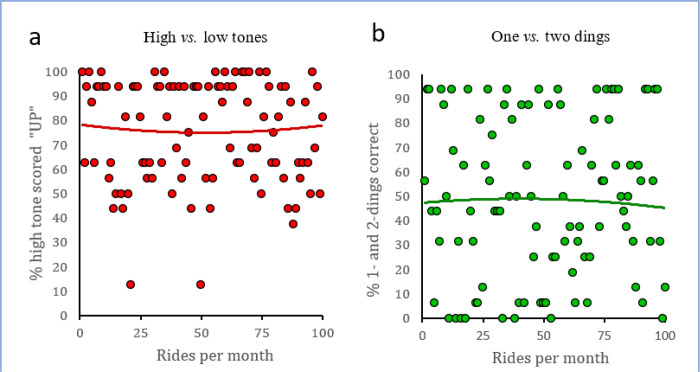
Correlation of elevator ridership with task performance. The percent of higher tone stimuli scored as “UP” and lower tone as “DOWN” (**red circles, A**) or percent of 1-and 2-ding stimuli scored correctly (**green circles, B**) is plotted for each subject in the second experiment against their reported elevator ridership. Solid lines are second order regression lines and demonstrate the absence of significant correlations between ridership and performance.

Taken together, these results offer no evidence of subjects having implicit knowledge of the relationship between ding-number and elevator direction, suggesting that over the course of these participants’ lives, information about the second ding was not effectively processed. This is even though half the participants in Experiment 2 reported that they rode elevators at least twice a month and a quarter reported riding one at least 7 times a month; indeed, individual ridership did not correlate with accuracy predicting elevator direction (Fig. 3).

### Experiment 3: Experiencing blindness

A third study clearly demonstrated that participants’ lack of knowledge of this elevator rule was not due to any inherent difficulty in determining this relationship=. After failing to correctly provide an answer to the Elevator Dilemma question, participants (*n* = 20) were taken to a bank of elevators and blindfolded. The experimenter called for elevators in both directions. Upon arrival of an elevator, the participant was asked which direction they thought the elevator was going and, after responding, was given feedback on whether they were right or wrong. This continued until the participant reported that they had learned that one ding indicated that an elevator was going up and two indicated that it was going down. Most participants solved the problem in fewer than 6 trials (median = 4, mean = 5.29, SD = 4.72, range = 2–20). Thus, when this auditory information was presented without the usual visual cues, participants rapidly deduced its significance, with most participants discovering the rule in four trials or fewer, suggesting that information such as that provided by the second ding (for descending elevators), though normally filtered out, is effectively attended to when needed.

## Discussion

As we navigate the world, our brain constantly filters our sensory inputs, selecting the information most relevant to our goals for subsequent attention, perception, and memory. In this real-world example of attentional filtering, not even one of over 1,100 participants had, over the course of their lives, recognized the fact that elevator direction is indicated by the sounds played by arriving elevators. This is despite on average taking elevators at least once every two weeks with 15% of our sample averaging more than once a day.

Why are so few people aware of this, despite often riding elevators daily? One possible explanation, based on Pavlov’s experiments on classical conditioning (3, *p. 283*) is the “blocking” effect of Kamin (4, *p. 283*), seen in rats when training on one stimulus precedes additional training that adds a second, simultaneously applied stimulus. Subsequent responses to the second, added stimulus are dramatically reduced compared to those seen when no training with the first stimulus alone precedes the two-stimulus training. Thus, training on the first stimulus alone *blocks* learning of the second, added stimulus. Kamin (4) concluded that surprise was critical for associative learning, a model made more explicit by Rescorla and Wagner’s (5) prediction error model. However, a relatively recent report described 15 failures to replicate a range of prior findings of blocking effects (6), leaving its general validity in question.

A second possible explanation is “sensory gating,” whereby the brain response to the second of two identical auditory stimuli is severely diminished (7). Typically studied with two identical clicks presented 500 msec apart, the amplitude of the P50 evoked brain response potential (ERP) produced in response to the second click is reduced by as much as 86% (8). A reduction of 47% is still seen for interstimulus intervals as long as two seconds (8), twice the interval used in our Elevator Dilemma task. This, arguably, would be adequate to suppress any post-thalamic attention to the second tone. Whether both or only one of these mechanisms in involved, their effect is an almost complete blockade of functional awareness of the significance of the second ding.

Attending to salient sensory cues while selectively filtering out irrelevant and redundant information is a highly adaptive behavior that comes with a cost. Here we have presented an example of the erroneous filtering of valuable information in a real-world paradigm, illustrating both the brain’s remarkable ability to filter out sensory stimuli calculated to be of little or no value, and its ability, in at least one case, to consistently filter out valuable information.

## Methods

### Informed consent

**was obtained from all subjects and/or their legal guardian(s)**, For Experiments 1 and 2, participants were consented with an online informed consent form. For Experiment 3, all participants were consented with a printed informed consent form. Both informed consent forms were approved by the Beth Israel Deaconess Medical Center Committee on Clinical Investigations. All experiments were performed in accordance with relevant guidelines and regulations as stated in the consent forms; **all experimental protocols were approved by the Beth Israel Deaconess Medical Center Committee on Clinical Investigations.**

## Experiment 1

### Participants and recruitment

Participants (*n* = 1,039, 18–50 years old)] were recruited through Amazon’s Mechanical Turk (2). They had to reside in the United States, have a Mechanical Turk HIT approval rate > 98, and have > 1,000 approved HITS (9).

### Protocol

Potential participants were invited to participate in “a research study to investigate human knowledge and beliefs.” They were asked for their race, gender, and ethnicity, and then given the online Elevator Dilemma questionnaire.

### Elevator Dilemma questionnaire

“Compared to other people, how would you rank yourself, from “much less” (1) to “much more” (8) on these questions:

- How perceptive do you think are compared to others?
- How much do you notice your environment compared to others?
- How curious are you compared to others?
- How creative are you compared to others?

Imagine that you are alone in a building, waiting for an elevator while blindfolded. Without removing your blindfold, would you be able to tell whether an arriving elevator was going up or down (yes/no)?”

If participant answers ‘Yes’:

“How would you know?“ (Free-write text box)

‘Which of these methods comes closest to the answer you gave:” (radio-button forced choice)

- I would ask someone waiting for or on the elevator.
- I would hear a recorded message on the elevator.
- I would detect a subtle difference in pressure based on whether the elevator came up or down to my floor.
- The “ding” announcing the elevator’s arrival would be higher pitched if it were going up.
- The elevator would ding twice if it were going down.
- I would just get on and know when it started to move.
- None of these

If participant answers ‘No’:

“Which of these do you think would be most likely to work:” (radio-button forced choice)

- I would ask someone waiting for or on the elevator.
- I would hear a recorded message on the elevator.
- I would detect a subtle difference in pressure based on whether the elevator came up or down to my floor.
- The “ding” announcing the elevator’s arrival would be higher pitched if it were going up.
- The elevator would ding twice if it were going down.
- I would just get on and know when it started to move.
- None of these seems likely.

All participants: “And finally, how confident are you of your answer?”

- Just guessing
- Not very confident
- So-so
- Confident
- Very confident Certain

## Experiment 2

### Participants and recruitment

Participants (*n* = 150, age 18–50) were recruited through Amazon’s Mechanical Turk (2). Only 115 of the 150 (77%) completed the protocol, the remainder quitting early because of the amount of time required. All 150 were invited to participate in “a research study to investigate human knowledge and beliefs,” and had to reside in the United States, have a Mechanical Turk HIT approval rate > 99, and have > 1,000 approved HITS (9).

### Tones

Stimuli consisted of four recorded elevator sounds. The high (C_6_ – 1,046 Hz) and low (F_4_ – 349 Hz) tones, as well as the single-ding tone (F_5_ – 698 Hz) were 1,000 ms in duration. The two-ding sound consisted of two tones (F_5_ – 698 Hz) of 1,000 ms duration, separated by 500 ms of silence.

### Protocol

Participants were asked for their age and gender, and then given a modified version of the Elevator Dilemma questionnaire, in which they were only asked whether they could tell which way an elevator was going if blindfolded and, if they answered “yes,” how they could tell. While half the subjects (57 out of 114) answered “yes,” none correctly reported that they could tell by whether the elevator dinged once or twice.

After answering these questions, they were presented with two sets of 16 trials each in which they heard recorded elevator sounds and were asked whether the sounds indicated that the approaching elevator was going up or going down. In the first set, 8 trials consisted of recordings of a single ding, and 8 had two dings. In the second set, all trials contained only a single ding, but 8 were of a higher pitch (C_6_ – 1,046 Hz) than the other 8 (F_4_ – 349 Hz).

## Experiment 3

### Participants and recruitment

Participants were recruited through postings to local university job boards. To qualify, potential participants had to confirm that they (*i*) had a US social security number (for payment), (*ii*) were between 18 and 25 years old, (*iii*) had a normal bedtime between 22:00 and 2:00, (*iv*) typically obtained 6 and 10 hours of sleep per night, (*v*) consumed less than 3 caffeinated beverages per day and 5 alcoholic drinks per week, and (*vi*) had no psychiatric, neurological, or sleep-related disorders and were taking no psychoactive medications. They also had to agree (*vii*) to keep a “regular” sleep schedule for 3 nights and (*viii*) to abstain from any psychoactive drugs for 24 hrs prior to the study.

#### Protocol

Participants completed the Elevator Dilemma questionnaire used in Experiment 1. They then completed the Chapman Scales for Perceptual Aberration (10), Magical Ideation (11) and Social Anhedonia (12) and were then tested for sensory gating (7). Results from the Chapman scales and sensory gating test are not reported on here.

Participants were then taken to the elevator lobby on the eighth floor of the Feldberg Building at BIDMC and blindfolded. The researcher then pressed the up and down call buttons repeatedly in a pseudorandom order. Each time an elevator arrived, the participant was asked whether they thought it is going up or down. After they gave their answer, the participants were told which way the elevator was, in fact, going. Participants were instructed that the trials would stop once they could correctly state how to tell which way an elevator was going or after 20 trials had been completed.

## Figures and Tables

**Table 1.** Responses of participants who answered that they knew the answer to the Elevator Dilemma and then selected that “the elevator would ding twice if it were going down” (n = 3).

	Please describe in detail how you would explain to a friend how they could know which way an elevator is going.	How do you know this works?
**X**	“The closer the sound the elevator appears to be, the more likely it’s going down”	“I’m just guessing”
**X**	(no answer given)	(no answer given)
**?**	“I recently learned that elevators make one tone for up and another for down.”	“Observation & experience riding elevators.”

## Data Availability

All data are available on request from rstickgold@hms.harvard.edu.
